# The importance of taking ART appropriately in children and adolescents with HIV-1 to reach the highest capacity of immune function later in life

**DOI:** 10.3389/fimmu.2022.860316

**Published:** 2022-07-27

**Authors:** Katrine Schou Sandgaard, Triantafylia Gkouleli, Teresa Attenborough, Stuart Adams, Deena Gibbons, Mette Holm, Sarah Eisen, Helen Baxendale, Anita De Rossi, Savita Pahwa, Benny Chain, Athina S. Gkazi, Nigel Klein

**Affiliations:** ^1^ Infection, Immunity and Inflammation, University College London (UCL) Great Ormond Street Institute of Child Health, London, United Kingdom; ^2^ Department of Pediatrics and Adolescent Medicine, Aarhus University Hospital, Aarhus, Denmark; ^3^ University College London (UCL) Zayed Centre for Research into Rare Disease in Children, London, United Kingdom; ^4^ Genetics and Rare Diseases, Great Ormond Street Hospital for Children, London, United Kingdom; ^5^ Peter Gorer Department of Immunobiology, Kings College London, London, United Kingdom; ^6^ Tropical Diseases, University College London Hospital, London, United Kingdom; ^7^ Clinical Immunology Department, Royal Papworth Hospital, Cambridge, United Kingdom; ^8^ Department of Mother and Child Health, University of Padova, Padova, Italy; ^9^ Sylvester Comprehensive Cancer Center, University of Miami, Miami, FL, United States; ^10^ University College London (UCL) Division of Infection and Immunity, University College London (UCL) Cruciform Building, London, United Kingdom

**Keywords:** HIV-1, children, immune reconstitution, thymic output, antiretroviral therapy (ART), T cell receptor repertoires, T cell receptor clonal expansions, high throughout sequencing

## Abstract

Current antiretroviral therapy (ART) guidelines recommend treating all children with HIV-1 infection. This has changed from the broader use of ART to treat children to improve morbidity and minimise mortality. However, prior to current recommendations, not everyone with HIV-1 received timely treatment. What happens to the paediatric immune system when HIV-1 replication is not appropriately supressed remains unclear. 11 samples from adolescents with HIV-1 on ART and uninfected controls in the UK, aged 12–25 years, were examined; overall, adolescents with CD4^+^ counts > 500/μl and a viral load < 50 copies/ml were compared with adolescents with CD4^+^ counts < 500/μl and a viral load > 50 copies/ml at time of sampling. Measurements of thymic output were combined with high throughput next generation sequencing and bioinformatics to systematically organize CD4^+^ and CD8^+^ T cell receptor (TCR) repertoires. TCR repertoire diversity, clonal expansions, TCR sequence sharing, and formation of TCR clusters in HIV-1 infected adolescents with successful HIV-1 suppression were compared to adolescents with ineffective HIV-1 suppression. Thymic output and CD4^+^ T cell numbers were decreased in HIV-1 infected adolescents with poor HIV-1 suppression. A strong homeostatic TCR response, driven by the decreased CD4^+^ T cell compartment and reduced thymic output was observed in the virally uncontrolled HIV-1-infected adolescents. Formation of abundant robust TCR clusters and structurally related TCRs were found in the adolescents with effective HIV-1 suppression. Numerous CD4^+^ T cell numbers in the virally controlled adolescents emphasize the importance of high thymic output and formation of robust TCR clusters in the maintenance of HIV-1 suppression. While the profound capacity for immune recovery in children may allow better opportunity to deal with immunological stress, when ART is taken appropriately, this study demonstrates new insights into the unique paediatric immune system and the immunological changes when HIV-1 replication is ongoing.

## Introduction

Current antiretroviral therapy (ART) guidelines recommend treating all children with HIV-1 (1). Recommendations have moved from reducing short-term morbidity and mortality of children with HIV-1 towards optimizing immune status to provide immune protection into adulthood (2, 3). However, the impact of the timing of ART introduction to children remains less clear, and in resource-poor settings, many people currently living with HIV-1 are not taking their ART as recommended or find it difficult to do so due to lack of appropriate formulation availability. Children with HIV-1 have a profound capacity for immune recovery following ART due to their high thymic output, providing a reasonable T cell output is maintained (3–9). The thymus is sensitive to events such as HIV-1 infection although it does have a great capacity for endogenous repair (10–12). Thymic output peaks at one year of age, declining to much lower levels by early adulthood (13, 14). In contrast to adults, who rely on peripheral T cell proliferation of existing T cell clones, the unique immune capacity of children allows the establishment of a highly diverse naïve T cell population (9, 11, 13–15). However, the capacity to fully recover from immunological damage can be lost in children alongside maintaining viral control and a high CD4^+^ T cell count (1, 3). It is well known that HIV-1 damages the T cell receptor (TCR) repertoire diversity in children and particularly in adults with a depletion of shared and structurally related TCRs (16–19). The TCR repertoire has been shown to be heavily biased in HIV-1 individuals with expanded clonotypes perhaps contributing to the persistence of the HIV-1 reservoir (20). Measurements of TCR repertoires and functional TCR clonotypes in children with HIV-1 is highly relevant in finding a cure against HIV-1. Clarifying mechanisms to specifically boost and maintain a high TCR repertoire and cytotoxic T cells with high affinity for HIV-1 infected T cells may contribute to a therapeutic vaccine approach (21).

Some HIV-1-infected patients receiving ART through prior guidelines have persistently low CD4^+^ T cells despite sustained viral suppression, so-called poor immunological responders. Their early identification is important due to their higher morbidity and mortality (22, 23). In contrast, HIV-1-infected individuals may control viral replication and maintain CD4^+^ T cell numbers for long periods, even despite deferred ART (24–27). In this study, we identified five groups. A) patients with HIV-1 who received ART before the age of 5 years with efficient viral control and CD4^+^ counts > 500/μl, B) patients with HIV-1 receiving ART after 5 years of age with efficient viral control and CD4^+^ counts >500/μl, C) patients with HIV-1 receiving ART after 5 years of age with poor viral control and CD4^+^ counts < 500/μl, D) horizontally infected HIV-1 patients with efficient viral control and CD4^+^ counts > 500/μl, and E) HIV-1 uninfected controls. We have measured thymic output and used high throughput TCR sequencing and bioinformatic techniques to characterize the TCR repertoire. We have obtained novel insight into the development and function of the adaptive immune system in groups with different responses to ART. The low sample size of this study is a limitation that precludes definitive statistically analysis, hence the results and conclusions will require validation in a larger cohort.

## Materials and methods

### Participants and samples

*Peripheral blood mononuclear cells (*PBMCs*)* from vertically and horizontally infected children and young adults and uninfected controls in the UK, all aged 12–25 years, had been acquired as part of a vaccine study investigating the development of humoral immunity (28). Patient characteristics are summarized in **Table 1**. Healthy young adults with no immunological disease history formed the control group and no uninfected children aged 12 years were included in the study, which is the reason why these individuals are 21-22 years of age. For our sub study, eligibility criteria included being on any ART regimen at the time of blood sampling and having at least 5 million viable PBMCs. For each group the following criteria had to be met: A) vertically infected patients initiating ART < 5 years of age with CD4^+^ counts > 500/μl and a viral load < 50 copies/ml at time of sampling, B) vertically infected patients receiving ART > 5 years of age with CD4^+^ counts > 500/μl and a viral load < 50 copies/ml at sampling, C) vertically infected patients receiving ART > 5 years of age with CD4^+^ counts < 500/μl and a viral load > 50 copies/ml at sampling, D) Horizontally infected patients on ART with CD4^+^ counts > 500/μl and a viral load < 50 copies/ml at sampling, and E) HIV-1 uninfected controls (**Table 1**). Two patients met the eligibility criteria in group A, two in group B, three in group C, two in group D, and two healthy controls were chosen in group E. The study was approved by the South Central – Hampshire A Research Ethics Committee REF number: 17/SC/0218.

**Table 1:** Patient characteristics.

**Group**	A	A	B	B	C	C	C	D	D	E	E
**Patient ID**	1	2	3	4	5	6	7	8	9	10	11
**Age in years (months)**	13 (4)	12 (9)	19 (8)	18 (7)	14 (3)	18 (11)	15 (3)	21 (11)	25 (7)	21 (11)	22 (1)
**HIV transmission **	Vertical	Vertical	Vertical	Vertical	Vertical	Vertical	Vertical	Horizontal	Horizontal	N/A	N/A
**On ART**	yes	yes	yes	yes	yes	yes	yes	yes	yes	N/A	N/A
**Age when ART was started, years (months)**	2 (10)	4 (9)	13	13 (11)	10 (4)	10 (4)	5 (4)	15 (8)*	22 (7)*	N/A	N/A
**CD4+ count/μl**	1190	840	890	740	220	160	320	800	590	920	924
**Sex**	F	F	F	F	M	M	F	F	M	M	M

*Date of first positive test. N/A, not applicable.

### Fluorescence-activated cell sorting of T cell subpopulations

Total PBMCs were thawed and washed in RPMI 1640 medium (Invitrogen) containing 10% fetal calf serum (StemCell Technologies), 2 mM L-glutamine (Sigma), 100 U penicillin and 100 μg/ml streptomycin (Invitrogen) (complete medium, CM). Thawed PBMCs underwent FACS to isolate CD4^+^ cells and CD8^+^ cell subsets for downstream high throughput sequencing with T cell specific primers. The antibodies used were CD4-APC (BD-Biosciences) and CD8-APC-Cy7 (BD-Biosciences), and with a fixable live/dead stain (AQUA, Invitrogen). PBMCs were fixed using Cell Fix (BD-Biosciences). A CD3^+^ antibody was excluded since only T cell specific primers were used in the downstream analysis. Samples were analysed on a FACSAria III cell sorter using FACSDiva software v.8.0. The purity of each separated cell group was >95% comparing the number of cells sorted with the Flow analysis and number of cells pre-sorting. Sorted cell numbers varied between 200.000-1.000.000 cells. The variation in cell numbers was accounted for in the bioinformatic analysis subsampling all TCR reads down to the same number as described below.

**Figure 1:**
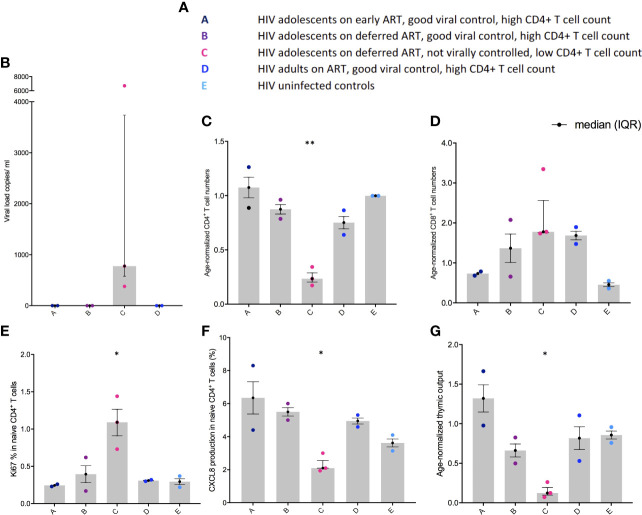
Viral load, age-normalised CD4^+^ and CD8^+^ T cell numbers, CXCL8 T cell production, and thymic output in adolescents with HIV-1. **(A)** Overview of the different groups. **(B)** Viral load (copies per ml). **(C)** Age-normalised CD4^+^ T cell counts. **(D)** Age-normalised CD8^+^ T cell counts. **(E)** Ki67 production in naïve CD4^+^, CD45RA^+^, CD31^+^ T cells (%). **(F)** CXCL8 production in naïve CD4^+^, CD45RA^+^, CD31^+^ T cells (%). **(G)** Thymic output (number of recent thymic emigrants measured by the mathematical model). Black lines: median(IQR). *P ≤ 0.05, **P≤ 0.005.

For all flow cytometry datasets, the gating strategy used forward scatter and side scatter to define our cell population and to exclude debris. Duoblets were removed before choosing live cells for further quantification of all the antibodies using two parameter density plots. FMOs and unstained controls were used in order to identify the positive dataset. Backgating was used to confirm gating strategies. For gating strategy see **Figure 1SA**.

### Cell stimulation for CXCL8 detection

Naïve T cells have an enhanced capacity to produce C-X-C motif chemokine ligand 8 (CXCL8), which is an important T cell effector function in human infants (29, 30). PBMCs were stimulated with phorbol myristate 13-acetate (PMA) (Sigma 10ng/ml) and ionomycin (1 μg/ml Sigma) in the presence of brefeldin A (BFA) (20 μg/ml Sigma) in CM for 3.5 hours at 37°C with 5% CO_2_ before intracellular stained with IL-8-AF488 (CXCL8). A negative control for CXCL8 was used with BFA only. The CXCL8 cytokine production shown in the results section is from PMA/ionomycin stimulated PBMCs. The BFA only control showed by large the same pattern as the stimulated cells, however with decreased CXCL8 production as expected (not shown).

### Flow cytometry for surface and intracellular staining

PMA/ionomycin-stimulated PBMCs were stained using fixable AQUA live/dead stain (Invitrogen) and the following antibodies: CD3-BUV395 (Invitrogen), CD4-BV605 (BD Biosciences), CD45RA-PerCPVio700 (BD Biosciences), CD31-BV421 (BD Biosciences), CD45RO-PE (BD Biosciences), and CD8-APC-Cy7 (BD Biosciences) in FACS buffer (phosphate-buffered saline containing 0.2% bovine serum albumin and 0.02% sodium azide). PBMCs were fixed and permeabilised (Foxp3, eBioscience). The PBMCs were intracellular stained with Ki67-AF647 (BD Pharmigen, measured in un-stimulated PBMCs) and IL-8-AF488 (CXCL8, BD Biosciences, measured in stimulated PBMCs) and incubated in the dark on ice for 1 hour. Samples were analyzed on an LSRII (Becton Dickinson) using FACSDiva software v.8.0. Subsequent data analysis was performed using FlowJo software 10.4. For gating strategy see **Figure 1SB**.

### Real-time PCR for TRECs

Real-time quantitative polymerase chain reaction (qPCR) was carried out for T cell receptor excision circles (TRECs) and a human control gene, T cell receptor alpha constant gene (*TRAC*), on a TaqMan 7500 Fast system (Applied Biosystems) using 12.5 µl TaqMan Universal Master Mix and 5 µl DNA. SJ TRECs forward primer (5’-CAC ATC CCT TTC AAC CAT GCT-3’) and probe (5’-FAM-ACA CCT CTG GTT TTT GTA AAG GTG CCC ACT-TAMRA-3’) were those designed by Douek et al.  (31), while the reverse primer for SJ TRECs (5’-TGC AGG TGC CTA TGC ATC A-3’), together with the forward (5’-TCC CTT AGT GGC ATT ATT TGT ATC ACT-3’) and reverse (5’-AGG AGC CAG CTC TTA CCC TAG AGT-3’) primers and probe (5’-HEX-TCT GCA CGG GCA GCA GGT TGG-TAMRA-3’) for SJ KRECs and the oligonucleotides and probe for TRAC gene **(**forward 5’-TGG CCT AAC CCT GAT CCT CTT-3’, reverse 5’-GGA TTT AGA GTC TCT CAG CTG GTA CAC-3’ and probe 5’-FAM-TCC CAC AGA TAT CCA GAA CCC TGA CCC-TAMRA-3’) were designed in Sottinis laboratory using Primer Express software version 3.0 (Applied Biosystems) as previously described (32, 33). The PCR assay for the three genes with the same standard protocol consisted in a first step at 50°C for 2 min, an initial heating at 95°C for 10 min, followed by 45 cycles of denaturation at 95°C for 15 s and a combined primer/probe annealing and elongation at 60°C for 1 min. A standard curve was generated for TRECs and *TRAC* using serial dilutions of the TREC/KREC/*TRAC* plasmid kindly provided by Sottinis laboratory. The number of TRECs per cell was calculated using the copy number ratio of TRECs : *TRAC* and multiplying by 2 to account for the presence of two copies of the *TRAC* gene in a diploid cell.

### Modelling thymic output

A model-based method for estimating thymic output and its validation using thymus size and cellularity from 0 to 20 years of age has previously been described in detail (14). The model was based on thymuses derived from patients between 0 to 20 years of age, but in the current study we have performed the mathematical model on group D and E too as done previously (6). In short, an explicit expression for thymic export in terms of total naïve cell numbers, naïve cell TREC content, and Ki67 expression is given as:


$$\begin{array}{c} \text{Thymic export }\left(cells/day\right)=\theta \left(t\right)\\ =\left(\frac{y\left(t\right)\tau \left(t\right)}{\Delta }+\frac{d\tau \left(t\right)}{dt}\right)\frac{N\left(t\right)}{c-\tau \left(t\right)} \end{array}$$

where *c* is a constant representing the average TREC content of thymic emigrants entering the peripheral naïve T cell population, *y*(*t*) is the flow cytometry-estimated Ki67 fraction of naïve CD4^+^ T cells, Δ is the duration of Ki67 expression, τ is the qPCR-estimated TREC concentration in the sorted naïve CD4^+^ T cell population, and *N*(*t*) is the total size of the naïve CD4^+^ T cell pool calculated from the clinical trial unit’s lymphocyte counts from fresh PBMC samples. Estimated total body CD4^+^, CD45RA^+^, CD31^+^ T cell numbers *N(t)* were calculated as previously described, referring to the linear relationship between blood volume and body weight (13, 34). Parameter values *c* and Δ were obtained as described previously (14).

### Genomic DNA and RNA isolation from FACS-sorted fixed cells

We developed a modified protocol for isolation of genomic DNA and RNA from fixed cells as previously reported (9, 35). Genomic DNA was extracted from low numbers (200,000-400,000) of CD4^+^ T cells using the DNA Microextraction Kit (QIAGEN). Briefly, cells were re-suspended in 100 μl buffer ATL and 10 μl proteinase K and incubated at 56°C for 1 hour and then 90°C for 1 hour to reverse the partial formaldehyde modification of the nucleic acids. Genomic RNA from sorted CD4^+^ and CD8^+^ T cells was extracted in two steps. In the first step, the formalin-fixed, paraffin-embedded tissue RNeasy kit (QIAGEN) was used. The cells were re-suspended in 150 μl buffer PKD, 10 μl proteinase K, and incubated at 56°C for 15 minutes and then at 80°C for 15 minutes. Next, the RNeasy Micro kit (QIAGEN) was used. RNA quantity and quality were verified on a Nanodrop and Qubit fluorometer (Thermo Fischer Scientific).

### TCR repertoire sequencing

TCR repertoire libraries were prepared using a RNA-based 3’ RACE protocol incorporating unique molecular identifiers for quantitative TCR sequencing. The unique molecular identifier (UMI)-based RACE protocol is now considered a gold standard. The method provides a template switch effect allowing continuous replication of the entire oligonucleotide. It captures all TCR variants present in the sample and only one primer set is required per reaction avoiding the use of multiple primer sets and thus any amplification bias. Full details of the method have recently been published (36–38). Between 200-500 ng RNA from FACS sorted CD4^+^ and CD8^+^ cells were used in the beginning of the TCR protocol for cDNA synthesis. Up to 12 final amplicon products were pooled together in the end and loaded, at 12pM concentration, on an Illumina MiSeq, using a version 2 chemistry 2x250PE kit.

### Data analysis

The FASTQ files produced on the MiSeq were processed using the Decombinator package (39, 40). Decombinator, which incorporates barcode dependent correction for sequencing error and PCR bias, annotates each TCR sequence according to V gene and J gene usage, and the highly variable complementary-determining region 3 (CDR3) sequence. The output from Decombinator is then grouped according to UMIs which is incorporated into each cDNA molecule by the ligation step. Within multiple identical TCR sequences in the same UMI group of equal abundance one is chosen arbitrarily and the rest discarded. The barcode information is essential since the probability of two identical TCRs having the same 12-mer barcode is very low. Identical TCR sequences in the same UMI group are discarded since they were most likely derived from the same TCR cDNA molecule by PCR/sequence error. The number of different UMIs paired with a single identical TCR sequence provides the frequency of the sequence in the sample.

The distribution of TCR abundances in each repertoire was summarised using the Gini coefficient, a measure of distribution inequality. The scale ranges from 0 to 1, where 0 = completely equal (x clones, all with identical frequencies) and 1 = completely unequal (i.e., tending towards sample oligoclonality). Because the Gini coefficient is affected by total population size and due to variation in numbers of cells sorted and RNA extracted, all repertoires were subsampled to the same number of reads at 5000 TCR sequences. The ratio between CD4^+^ cells/CD8^+^ cells sorted was 0.6. This was accounted for in the subsampling analysis. Gini indices were computed using the ineq package (version 0.2.13) in the R environment (version 3.3.2). Minimum subsampling depth for each sample was algorithmically determined to preserve sample distribution as recently published (38).

In response to antigens, structurally related TCRs expand in clusters with identical continuous amino acids (AA) in their sequences (motifs) (41, 42). First, the presence of TCR clusters using a shared triplet metric of similarity was computed as previously described (43). The algorithm returns clusters of enriched motifs of three AA, presenting the TCRs as nodes in a network. Second, the Hamming distance metric was computed visualizing the number of AA differences between TCRs. The model examines two pairwise TCRs of equal length at a time creating a TCR similarity network. A Hamming distance of 1 reflects a single AA change between two neighboring TCRs. Third, we examined which TCRs are predicted to bind the same MHC-restricted peptide antigen utilising the algorithm “Grouping Lymphocyte Interactions by Paratope Hotspots” (GLIPH) (42). GLIPH returned lists of significant, locally enriched motifs of four continuous amino acids that were more than 10-fold enriched in our T cell population. The T cell population was compared with a repeat random sampling of a large database with naïve T cell receptor CDR3s from 12 healthy individuals at the same sequencing depth. In our analysis, only continuous 4mer motifs was searched for, excluding the first three and last three amino acid residues in the CDR3 because they are not observed to be in contact with antigens, as recently published (42). GLIPH then searched for and returned TCR groups that are predicted to bind the same MHC-restricted peptide antigen combining global and local TCR sequence similarity, structural peptide antigen contact propensity, V-segment bias, CDR3 length bias and clonal expansion bias.

The R package ggplot2 (version 2.2.1) and Prism (version 8) were used for all data visualisations. All manual data analyses were executed using custom scripts written in R (version 3.3.2) and Python (version 3.7.3). The Decombinator package v3.1 was executed using Python (version 2.7.3). All software is freely available at https://github.com/innate2adaptive/Decombinator. The raw FastQ files are deposited at the Sequence Read Archive (https://www.ncbi.nlm.nih.gov/sra with accession to the SRA data: PRJNA797937).

The statistical test used for comparisons between virally supressed HIV-1 adolescents with CD4^+^ counts > 500/μl in group A and B compared to group C (HIV-1 adolescents with unsuppressed viremia and CD4^+^ counts < 500/μl) was an unpaired, non-parametric *t*-test assuming no Gaussian distribution. The low numbers of patients included was a limitation, which precludes a clear and statistically significant conclusion.

## Results

### Thymic output is highest in virally controlled HIV-1 children and adolescents

The five clinical groups are shown in **Figure 1A**. Viral load was higher in group C compared to the other groups in whom HIV-1 was controlled (**Figure 1B**). In parallel, age-normalised CD4^+^ T cell numbers were significantly lower (P < 0.0026), whereas age-normalized CD8^+^ T cell numbers were slightly higher (not significant (ns)) in group C compared to the virally supressed HIV-1 adolescents in group A and B (**Figures 1C, D**). Age normalised T cell numbers were calculated from published age-matched lymphocyte population reference values and y axes in **Figures 1C**, **D**, **G** shows the patients T cell number/expected T cell number for age (44). The reference values are previously published and may not represent the exact true number, which could be why group E seems low in **Figure 1D**. The nuclear protein, Ki67, measures proliferation as it accumulates in dividing cells during S, G2, and M phases during the cell cycle followed by degradation upon cell cycle exit (45). Ki67^+^ naïve CD4^+^ T cells were significantly higher in group C (P < 0.0444) compared to the adolescents supressing HIV-1 in group A and B (**Figure 1E**). CXCL8 production (%) in the naïve CD4^+^ T cells was significantly lower in group C (P < 0.0192) compared to the adolescents suppressing HIV-1 in group A and B, with a decreasing trend from group A to E (**Figure 1F**). Age-normalised thymic output measurements (estimated by the mathematical model) were significantly lower in group C (P < 0.0387) compared to the adolescents suppressing HIV-1 in group A and B, with the youngest virally controlled HIV-1 adolescents reaching the highest values (**Figure 1G**).

### Poor HIV-1 control drives increased clonal expansions in CD4^+^ and CD8^+^ T cells

**Figure 2:**
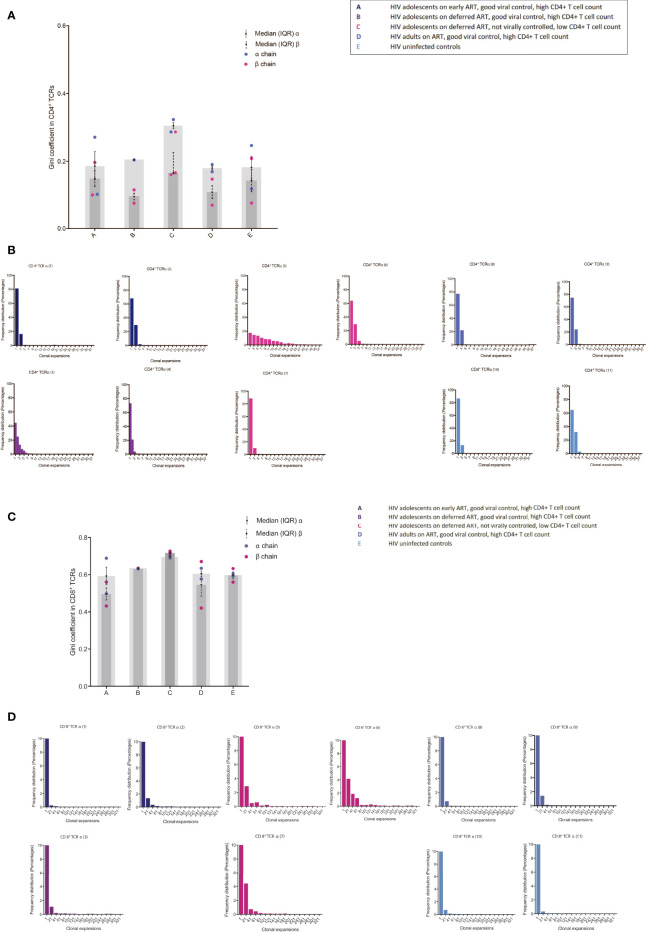
TCR abundance distribution in CD4^+^ and CD8^+^ T cells in adolescents with different responses to HIV-1 **(A)** The degree of clonal expansion in the CD4^+^ TCR repertoire measured using the Gini coefficient in TCR alpha (blue) and TCR beta sequences (pink). **(B)** Individual TCR sequence abundance distribution in the CD4^+^ TCR repertoire. **(C)** The degree of clonal expansion in the CD8^+^ TCR repertoire measured using the Gini coefficient in TCR alpha (blue) and TCR beta sequences (pink). **(D)** Individual TCR sequence abundance distribution in the CD8^+^ TCR repertoire. Black lines: median(IQR).

The Gini coefficient of the CD4^+^ TCRs was higher (ns) in group C compared to the other groups, suggestive of clonal expansions (**Figure 2A**). The α chain Gini index was consistently greater than the beta chain - a reflection of the lower diversity of TCR α due to the absence of a D gene (**Figure 2A**). When subsampling all alpha and beta TCR chains together from all patients, a few Gini coefficients were excluded, one due to too few reads and one due to too many reads (one in group B and one in group C). The number of clonally expanded TCRs is reflected in the abundance distribution (number of times the same TCR sequence are observed) (**Figure 2B**). The clonally expanded TCRs in individual CD4^+^ TCR repertoires were higher, particularly in one patient in group C, compared to the other individuals, most likely reflecting a higher proportion of effector/memory CD4^+^ T cells corresponding to a higher Gini coefficient as previously reported (**Figure 2B**) (9, 16). In the CD8^+^ TCRs, small Gini index increases were also observed in group C (ns) compared to the other groups but with less α/β chain differences (**Figure 2C**). The minor Gini index increase observed in group C was confirmed by a minor increase in the number of more abundant TCR sequences in group C compared to the other groups (**Figure 2D**). In general, in all the patients, higher CD8^+^ TCR Gini coefficients were observed than CD4^+^ TCRs reflecting a higher proportion of memory T cells in the CD8^+^ population as expected (9, 16). The frequency distribution goes above 90% in all the CD8^+^ TCRs (**Figure 2D**), and to visualize the clonal expansions, the threshold in the CD8^+^ figures were set to 10%.

### Poor HIV-1 control is associated with a specific and non-specific homeostatic clonal TCR response

**Figure 3:**
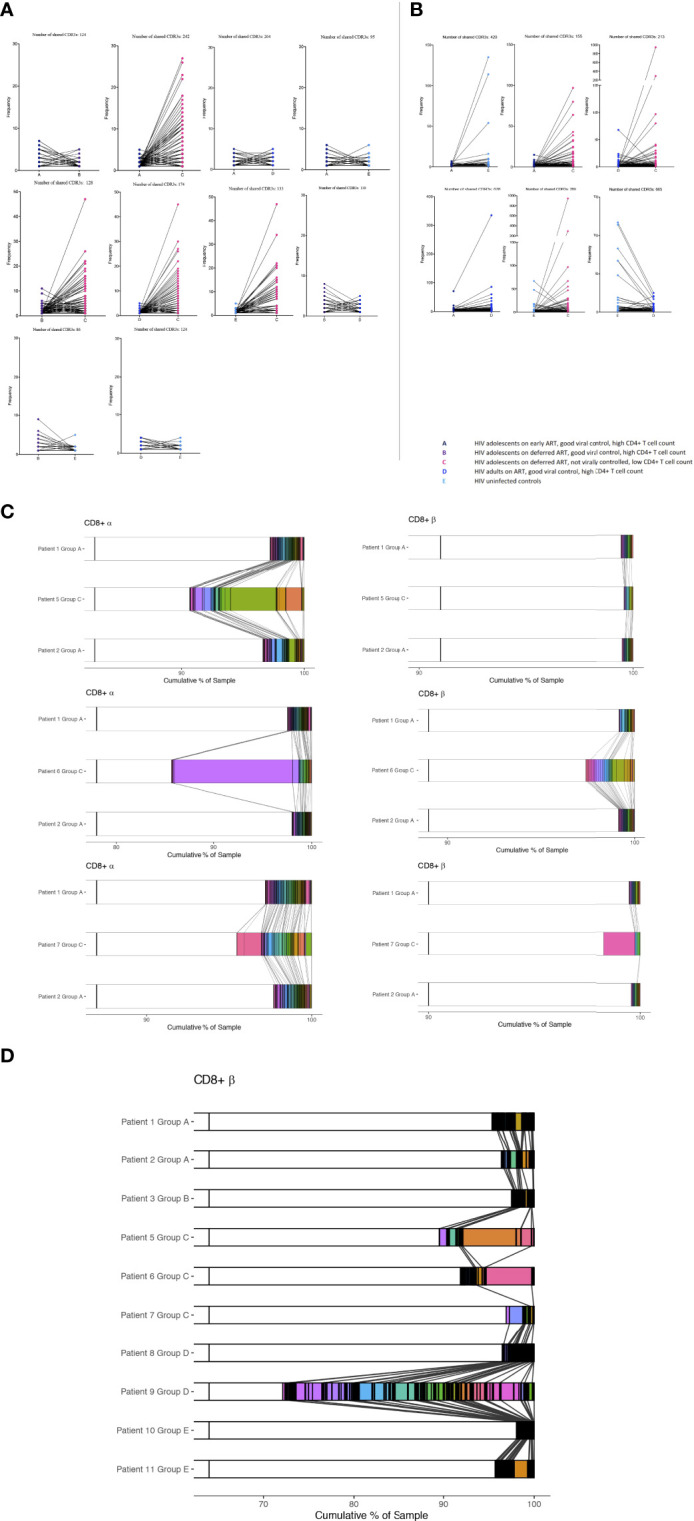
The abundance differences of the public TCR repertoire in children with different responses to HIV-1. The y axis shows the abundance of shared TCRs (dots) between groups. Lines are connecting identical shared TCRs between two groups. **(A)** CD4^+^ TCR repertoires and **(B)** CD8^+^ TCR repertoires. **(C, D)** Shared CDR3s between patients from CD8^+^ T cells are shown with connecting grey lines. Left panels show α chain samples; right panels show β chain samples. The horizontal bar represents a sample, with white space showing the percentage of CDR3s unique to that sample within each panel, and these constitute the majority of each sample. Within each of the six panels, all the CDR3s occurring in more than one sample are coloured and separated by black outlines, thus many CDR3s shared at low frequencies appear black. A broad coloured band indicates that the CDR3 has expanded multiple times. Within each panel, a given colour represents the same CDR3; grey lines overlaid show pairwise shared CDR3s.

We identified several CDR3 sequences that were shared between individuals, and the differences in abundances of these TCRs was visualized between groups (**Figure 3**). In the majority of the groups, identified shared sequences in the CD4^+^ TCR repertoire appeared to have low frequency abundances (frequency <10), reflecting mainly a naïve repertoire (9, 16). However, group C had profound increased abundances in their shared CD4^+^ TCR clones compared to all other groups (**Figure 3A**). The fact that the shared TCRs had increased abundances in group C compared to the HIV-1 uninfected controls suggests a random HIV-1 non-specific homeostatic clonal TCR response. The shared TCRs in the CD8^+^ T cell compartment included much higher abundancies, which most likely reflect the TCRs shifting into an effector/memory repertoire as expected (9, 16). Group C had higher abundances in some of their CD8^+^ TCR clones compared to the other groups; however, lower abundances were also observed in others (**Figure 3B**). Furthermore, it was observed that the virally controlled HIV-1 children in group A generally had lower CD8^+^ TCR abundances than the others, reflecting a more naïve CD8^+^ TCR repertoire, attesting to their higher thymic output and younger age (**Figure 3B**) (9, 16).

We then identified the percentage of each patient’s CD8^+^ CDR3s being shared between patients or being unique to the sample. The two samples from Group A are sequentially compared to the three samples from the Group C patients (**Figure 3C**). The shared CDR3s in group C show numerous clonal expansions compared to group A, attesting to their higher peripheral proliferation (Ki67) and low TCR repertoire diversity compared to the patients who are fully HIV-1 supressed. The same pattern was shown between groups B, D and E compared to group C (not shown). CDR3s from CD8^+^ T cells shared between *all* individuals in the study are shown in **Figure 3D**. Patient 4 was not included as there were not a sufficient number of reads for this analysis. HIV-1 patients who are fully suppressed are in general showing higher percentages of unique sequences with multiple shared CDR3s of low frequencies except patient 9 who was horizontally infected. Within sequences shared between all patients, group C had in general more expanded CDR3s. Shared CD4^+^ TCRs and TCRS shared between all patients in the α chain are shown in **Supplementary Figures 2SA-C**.

The expanded public (shared) TCRs included some sequences previously annotated as recognising EBV, HIV-1, and CMV; however, the TCRs shared between the HIV-1 adolescent with poor viral suppression (group C) and uninfected controls were not previously annotated, consistent with increased random HIV-1 non-specific homeostatic clonal expansions (**Table 2**).

**Table 2:** Public TCR sequences compared to VDJdb.

**CDR3**	**Epitope**	**Epitope.species**	**TCR**	**Chain**
CAGPGSQGNLIF	GILGFVFTL	InfluenzaA	CD8+	α
CAVMDSNYQLIW	NLVPMVATV	CMV	CD8+	α
CAVPYSGGGADGLTF	GILGFVFTL	InfluenzaA	CD8+	α
CAVSGYSTLTF	LLWNGPMAV	YellowFeverVirus	CD8+	α
CAVKDTDKLIF	YVLDHLIVV	EBV	CD8+	α
CAVNDYKLSF	LLWNGPMAV	YellowFeverVirus	CD8+	α
CAVRDSNYQLIW	NLVPMVATV	CMV	CD8+	α
CAASGGSYIPTF	LLWNGPMAV	YellowFeverVirus	CD8+	α
CAAGGSQGNLIF	GLCTLVAML	EBV	CD4+	α
CAVMDSNYQLIW	NLVPMVATV	CMV	CD4+	α
CAVSRGGSNYKLTF	TAFTIPSI	HIV-1	CD4+	α
CASNTGNQFYF	NLVPMVATV	CMV	CD4+	α
CAVGGSQGNLIF	GILGFVFTL	InfluenzaA	CD4+	α
CAVKDTDKLIF	YVLDHLIVV	EBV	CD4+	α
CAVRDSNYQLIW	NLVPMVATV	CMV	CD4+	α
CAVSSNDYKLSF	LLWNGPMAV	YellowFeverVirus	CD4+	α

### Poor HIV-1 control is associated with loss of TCR clusters and decreased TCR repertoire diversity

**Figure 4:**
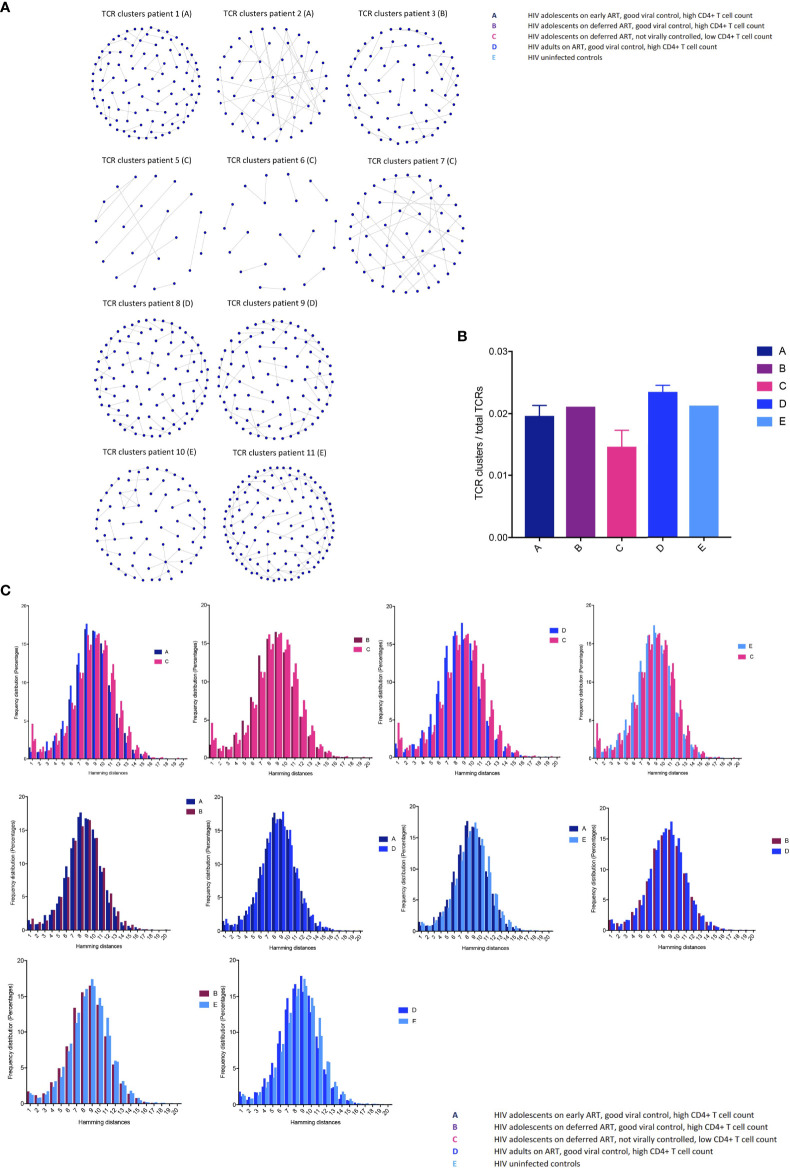
T cell receptor clustering in children with different responses to HIV-1 **(A)** Structurally related CDR3s with the same motifs in their sequences forming network clusters (shown as nodes) in the CD8^+^ T cell population. **(B)** TCR clusters (nodes) as a proportion of the number of total TCRs being clustered for each sample, showing median (IQR). **(C)** The distribution of pairwise Hamming distances between CDR3s in the CD8^+^ T cell repertoires.

Closely related TCRs have similar CDR3 regions with the same continuous amino acids (AA) in their sequences called motifs, observed to react to the same antigens (41, 42). To examine the presence of effective TCRs in response to antigens, we looked for TCR clusters (with the same motifs) across the groups. We used a shared triplet metric of similarity that returns clusters of enriched motifs of three amino acids as recently reported (9, 43). Clusters from each individual are visualised as nodes in a network, each node representing a CDR3. We observed a higher number of structurally related TCR clusters in the HIV-1 controlled adolescents and uninfected controls compared to group C in both the CD8^+^ (**Figure 4A**) and CD4^+^ TCR repertoires (**Figure 3S**). We measured the number of CD8^+^ TCRs in a cluster (the number of nodes in **Figure 4A**), as a proportion of the number of total TCRs being clustered for each sample. The metric is seen in **Figure 4B**; there is a decreasing trend in group C compared to the virally controlled adolescents with HIV-1 (P < 0.0559).

We then further surveyed for specific CDR3 structures such as AA sequence similarities using the Hamming distance metric in the CD8^+^ T cell compartment, because this population most likely reflected a memory population in our study (attesting to the high Gini coefficients in **Figure 2**). The model tests two pairwise CDR3s of equal length at a time and shows the number of AA differences between CDR3 sequences. A Hamming distance of 1 reflects a single AA change between two neighbouring CDR3s. The differences in Hamming distances were not major; however, the HIV-1 uncontrolled adolescents had greater frequencies of the lowest Hamming distances compared to the HIV-1 controlled patients, which suggests a lower TCR repertoire diversity (**Figure 4C**).

Finally, we examined clustering TCRs according to their CDR3 sequence similarity and motif conservation using GLIPH (42). GLIPH predicts which TCRs can recognise and respond to the same MHC-restricted peptide antigens. The algorithm yields several lists of significant, locally enriched motifs detected within thousands of CDR3s in each individual. We found a higher number of enriched amino acid motifs in the HIV-1 controlled adolescents and uninfected controls compared to group C (**Table 3**). The GLIPH algorithm then estimates the number of similar antigen specificity groups likely to react to the same antigens within the TCR repertoire (**Table 3**). Overall, we observed a lower number of specificity groups in group C, reflecting a decreased TCR repertoire diversity.

**Table 3:** TCR clustering characteristics in the CD8^+^ T cell population.

**Group**	A	A	B	C	C	C	D	D	E	E
**Patient ID**	1	2	3	5	6	7	8	9	10	11
**Numbers of significantly enriched local motifs**	304	352	264	214	219	166	276	270	274	321
**Numbers of significant antigen specificity groups**	769.167	823.545	345.558	110.021	124.010	29.153	452.807	357.882	311.012	714.301

## Discussion

This current study reports on the detailed immunological differences between children and adolescents receiving different ART strategies leading to different CD4^+^ T cell numbers and viral loads at time of sampling. We highlight how thymic output and a high TCR repertoire diversity are driving important TCR clusters to keep HIV-1 reservoirs suppressed.

In the current study, adolescents with poor HIV-1 control had significantly decreased thymic output, decreased CXCL8 production within the naïve CD4^+^ T cell population, increased homeostatic proliferation measured by Ki67, and profound changes in TCR repertoires, signifying the need for immune enhancement as previously described (22).

The increased clonal expansions in group C combined with increased proliferation by Ki67 in the CD4^+^ T cell repertoire suggests a homeostatic replenishment driven by the contracted CD4^+^ T cell compartment and decreased thymic output. The numerous shared TCRs between uninfected controls and group C with low HIV-1 viral control were seen to highly expand in group C, demonstrating a HIV-1 non-specific and random homeostatic replenishment of the T cell repertoire (46–48). Clonal expansions, perturbations in TCR repertoires and higher peripheral proliferation rates are compromising the repertoire diversity and implying premature immune ageing in the adolescents with poor viral control as previously described (9, 16, 19, 49–51).

We observed more TCR clusters and a higher number of enriched motifs and antigen specificity groups in the HIV-1 controlled children and adolescents compared to the HIV-1 adolescents lacking viral control. These results suggest that the formation of focused, structurally related TCR clusters against antigens are important in HIV-1 eradication in accordance with previous findings (24–27, 52). We found that some of the clonally expanded TCRs were likely to be specific to HIV-1, CMV, and EBV in agreement with a recent study, implying the persistence of CMVs and other latent infections that induces antigenic stimulation, which would favour the maintenance of HIV-1-infected cells in group C (20). This in turn may explain why group C cannot control HIV-1 due to their significant lack of important HIV-1 suppressive TCR clusters and structurally related TCRs. However, in relation to antigen specificity using the VDJdb, one single chain match does not conclude receptor specificity, which is a limitation to the current study. A rationale for the development of therapeutic strategies aimed at limiting antigen-driven proliferation during ART has recently been suggested to reduce the HIV-1 reservoir, which may be achieved by decreasing the antigen load of treatable pathogens such as CMV (20). The antigen driven proliferation could further be the result of inadequate adherence to ART. It is well known that cytotoxic T cell clonotypes with high affinity for HIV-1 infected cells is associated with HIV reservoir killing and the ability to boost and maintain these clonotypes are of high importance in finding a cure against HIV-1 (21). The lack of specific clonotype antigen specificity is a limitation to our study and functional studies of T cell reactivity will be required to confirm the antigen-specificity of the T cells responding to ART. Further, only 11 patients were included in the study with differences in age and sex in our small groups which is a limitation that precludes a clear and statistically significant conclusion.

In conclusion, our study documents differences in both CD4^+^ and CD8^+^ T cell repertoires between virally controlled HIV-1-infected - and virally uncontrolled HIV-1-infected children and young adults. The changes reflect a strong homeostatic, however both specific and HIV-1 non-specific response, driven by the contracted CD4^+^ T cell compartment and reduced thymic output in the adolescents with ongoing viral replication. The immunological advantages in the virally controlled adolescents highlight the importance of a high thymic output, diverse TCR repertoire and formation of robust TCR clusters in the maintenance of HIV-1 suppression. The capacity for immune recovery in children allow them a better opportunity to deal with immunological stress such as HIV-1, when HIV-1 replication is supressed (3, 4, 6–11). However, this present study demonstrates the importance of ART during childhood and adolescence, not only to increase survival, but to reach the highest capacity of immune function later in life.

## Data Availability Statement

The datasets presented in this study can be found in online repositories. The names of the repository/repositories and accession number(s) can be found below: https://www.ncbi.nlm.nih.gov/, PRJNA797937.

## Ethics Statement

The studies involving human participants were reviewed and approved by the South Central – Hampshire A Research Ethics Committee REF number: 17/SC/0218. Health Research Authority 2 Redman Place, Stratford, London, E20 1JQ. Written informed consent to participate in this study was provided by the participants’ legal guardian/next of kin.

## Author Contributions

KS performed the Flow cytometry and NGS experiments, applied the mathematical and bioinformatic analysis to the data, created the data presentation/visualization, and wrote the original draft. NK designed the study aims and acquired the financial support of the study. NK and AG oversaw and had leadership responsibility for the research planning and execution and contributed to drafting the manuscript. AG designed and supervised the NGS methodology. BC developed the NGS methodology. BC supervised the bioinformatics. TG contributed to performing NGS experiments. TA contributed to performing bioinformatics and designed the data visualization in **Figure 2**. SA designed and performed the TRECs analysis. DG designed the CXCL8 methodology. BC, TA, DG and TG contributed to drafting the manuscript. MH, SE, AR, SP and HB contributed to the conception and design of the study and writing the manuscript draft. All authors contributed to manuscript revision and read and approved the submitted version.

## Funding

This work was supported by Reuben Centre for Virology and Metagenomics, Action Medical Research, ViiV, UK Medical Research Council, United Kingdom, the Health Research Foundation of Central Denmark Region, Aarhus University Research Foundation, and the Lundbeck Foundation, Denmark. The funding source had no involvement in the study design, data collection, analysis, interpretation, or drafting of the paper. The corresponding author confirms that she had full access to all the data in the study and had final responsibility for the decision to submit for publication.

## Acknowledgments

Many thanks to Dr Ben Margetts, who wrote some initial scripts which were later adapted to produce some of the figures presented for this analysis.

## Conflict of Interest

The authors declare that the research was conducted in the absence of any commercial or financial relationships that could be construed as a potential conflict of interest.

## Publisher’s Note

All claims expressed in this article are solely those of the authors and do not necessarily represent those of their affiliated organizations, or those of the publisher, the editors and the reviewers. Any product that may be evaluated in this article, or claim that may be made by its manufacturer, is not guaranteed or endorsed by the publisher.
