# Supplementary material for: The importance of taking ART appropriately in children and adolescents with HIV-1 to reach the highest capacity of immune function later in life
Source: Front Immunol. 2022 Jul 27;13:860316. doi: 10.3389/fimmu.2022.860316 (PMC9364750; doi:10.3389/fimmu.2022.860316)
Supplement: Supplementary file 1 [file DataSheet_1.pdf]

Fig.1SA

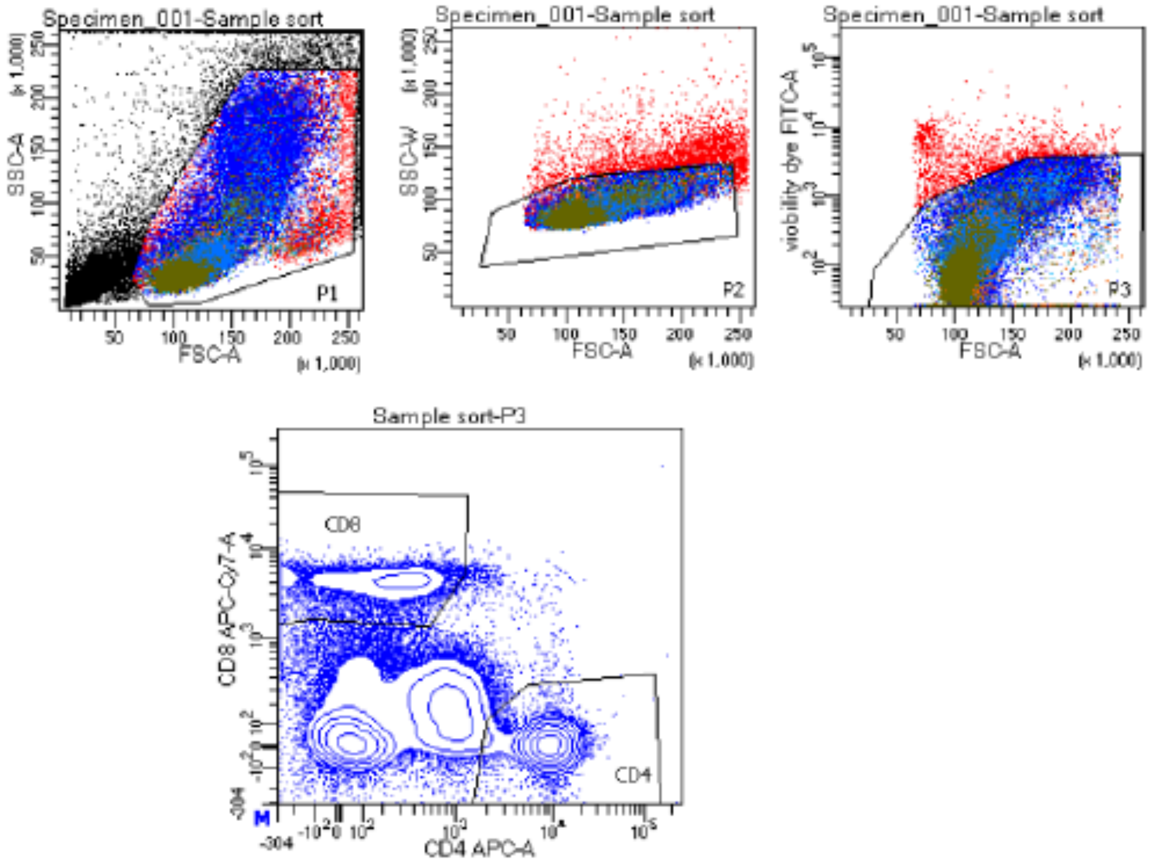

Gating strategy in FACS sorted T cells. For all flow cytometry datasets, the gating strategy used forward scatter and side scatter to define our cell population and to exclude debris. Duoblets were removed before choosing live cells for further quantification of all the antibodies using two parameter density plots. FMOs and unstained controls were used in order to identify the positive dataset. Backgating was used to confirm gating strategies

Fig.1SB

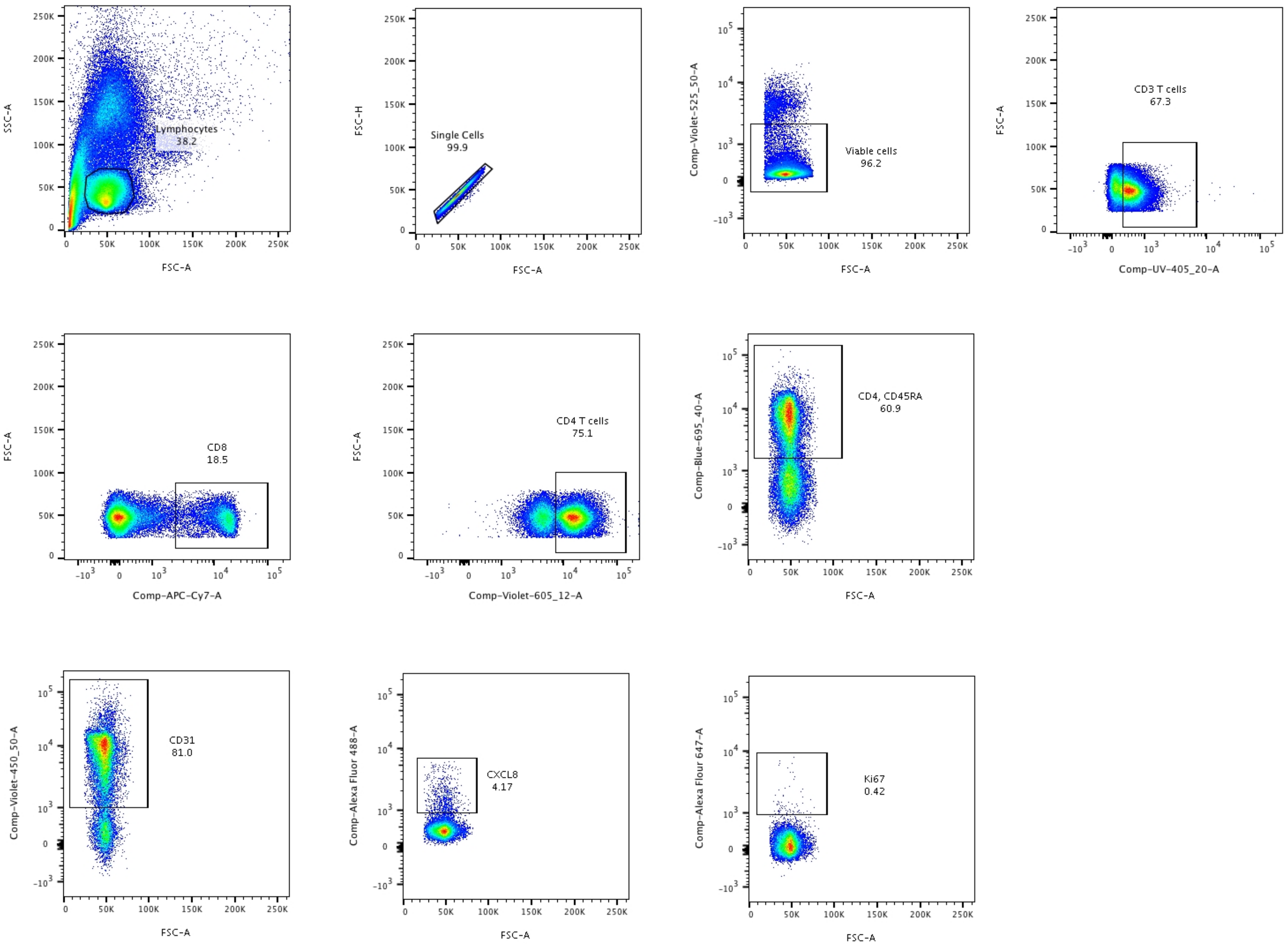

Gating strategy in the multicolour panel

Fig.2SA

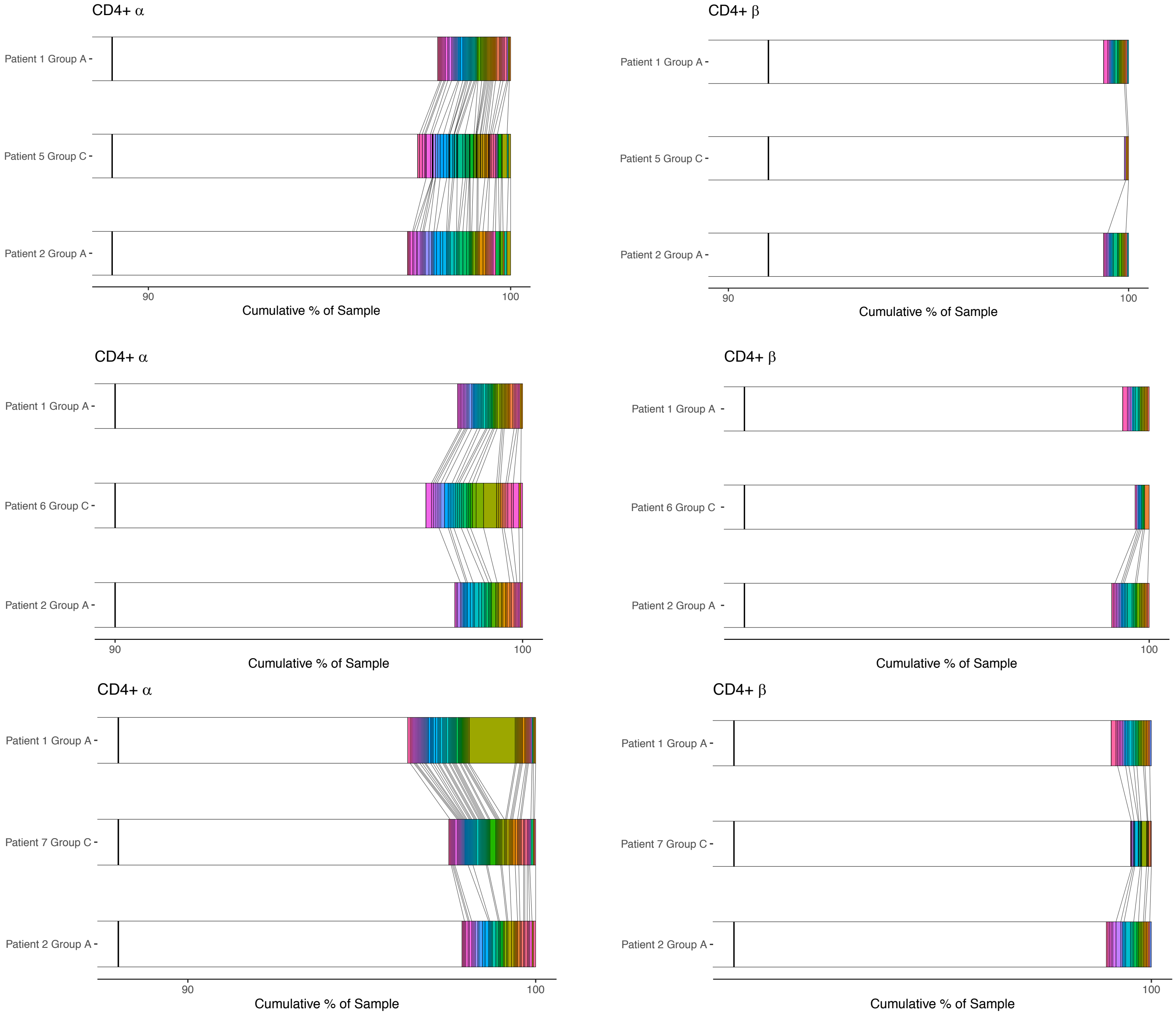

Shared CDR3s between patients from CD4⁺ T cells. The white horizontal bars represent the cumulative percentages of unique CDR3s and constitute the majority of each sample. All shared CDR3s have been colour-coded alphabetically and the narrow coloured lines (appearing black) represent CDR3s with very low frequencies. The same colour represents the same CDR3. A broad coloured band means that the CDR3 has expanded multiple times.

Fig.2SB

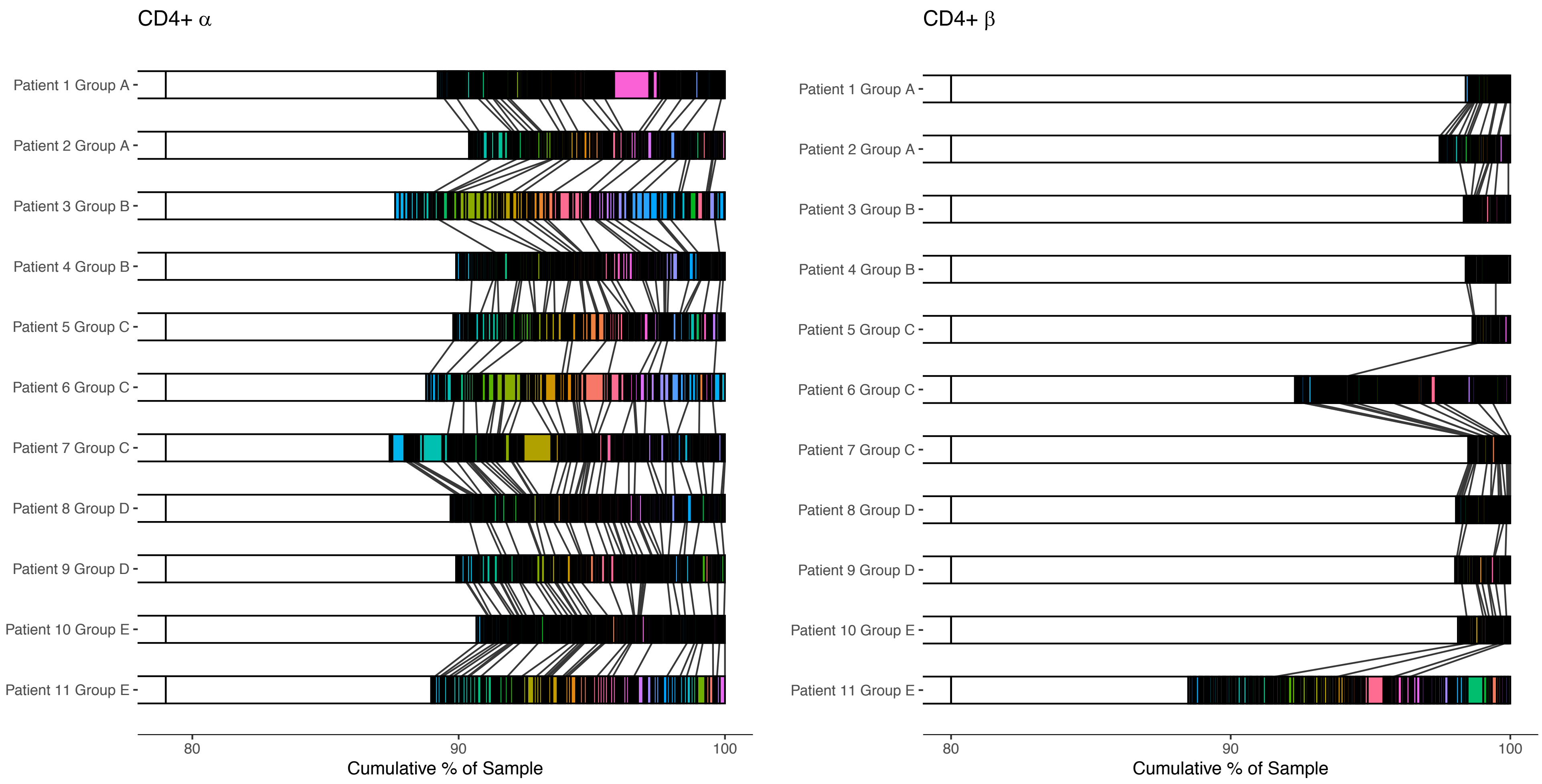

Shared CDR3s between patients from CD4$^+$ T cells. The white horizontal bars represent the cumulative percentages of unique CDR3s and constitute the majority of each sample. All shared CDR3s have been colour-coded alphabetically and the narrow coloured lines (appearing black) represent CDR3s with very low frequencies. The same colour represents the same CDR3. A broad coloured band means that the CDR3 has expanded multiple times.

Fig.2SC

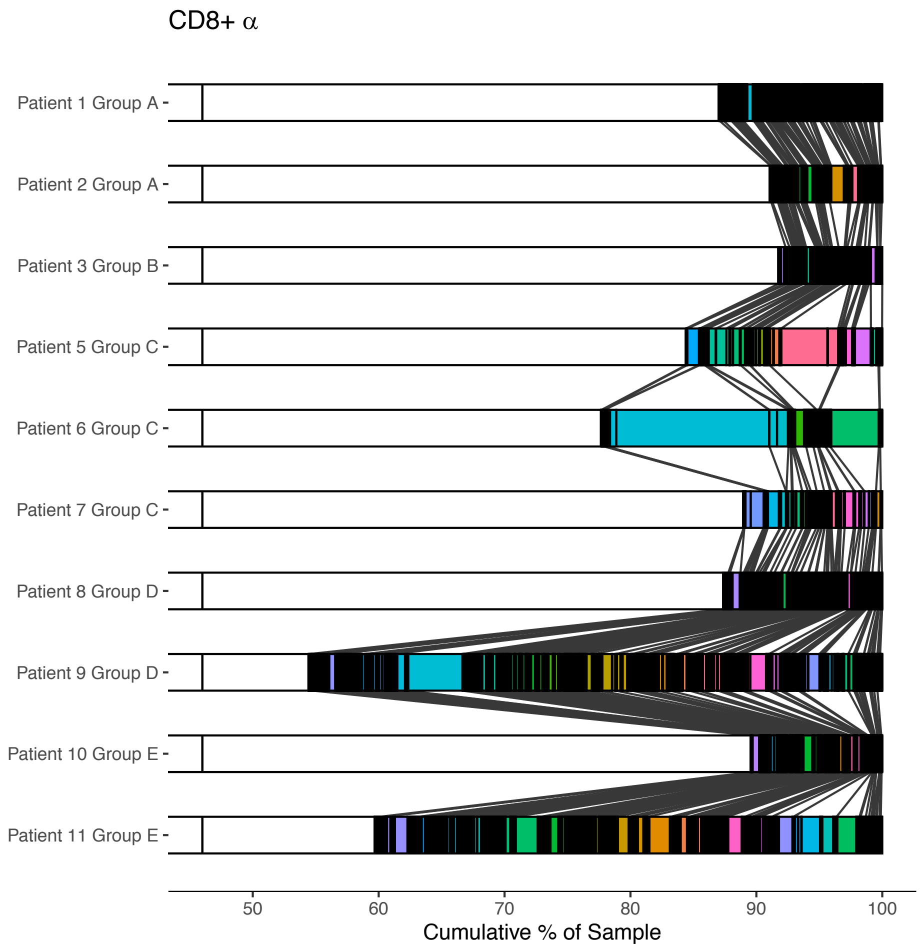

Shared CDR3s between all patients from CD8^{+} T cell alpha chains. The white horizontal bars represent the cumulative percentages of unique CDR3s and constitute the majority of each sample. All shared CDR3s have been colour-coded alphabetically and the narrow coloured lines (appearing black) represent CDR3s with very low frequencies. The same colour represents the same CDR3. A broad coloured band means that the CDR3 has expanded multiple times.

Fig. 3S

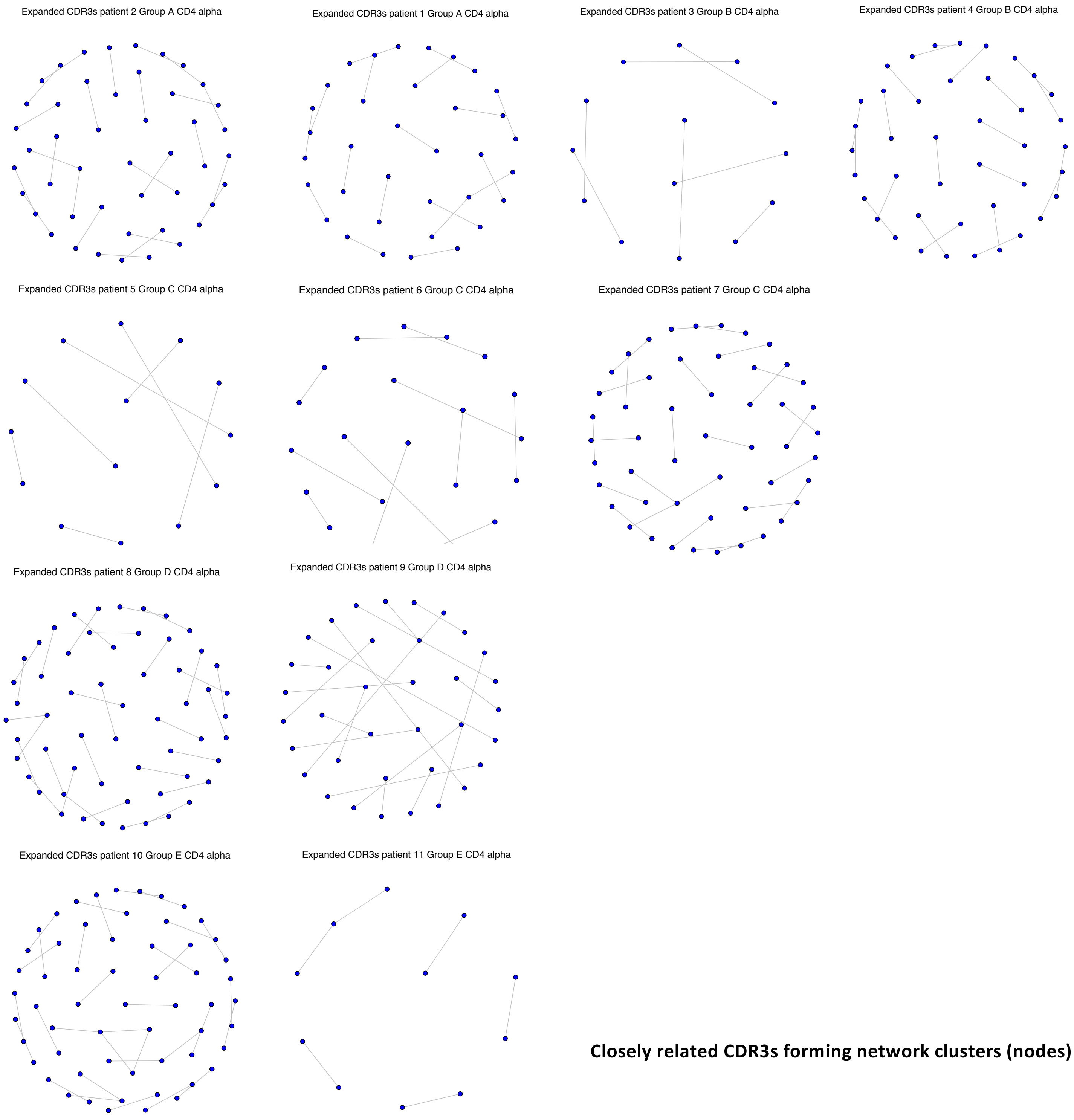

Closely related CDR3s forming network clusters (nodes) in the $CD4^{+}$ T cell population.
